# Effects of maneuver of hair-washing motion and gender on oxygen uptake and ventilation in healthy people

**DOI:** 10.1038/s41598-020-69945-5

**Published:** 2020-08-04

**Authors:** Miki Takahata, Masao Ishizawa, Takuya Uchiumi, Michiyasu Yamaki, Toshiaki Sato

**Affiliations:** 1 Graduate School of Health Sciences, Yamagata Prefectural University of Health Sciences, Yamagata, Japan; 2 Department of Occupational Therapy, Yamagata College of Medical Arts and Sciences, Yamagata, Japan

**Keywords:** Physiology, Health care

## Abstract

It is known that patients with chronic obstructive pulmonary disease experience dyspnea during unsupported arm exercise (UAE). We examined the respiratory variables in during a hair-washing motion for healthy young people requiring the UAE to find the effects across gender, motion, and speed. In this study, 33 healthy young people were enrolled. Participants performed the following four types of hair-washing motions: both hands with fast speed, both hands with slow speed, one hand with fast speed, and one hand with slow speed. The respiratory variables such as oxygen uptake (${\dot{\text{V}}}$O_2_), carbon dioxide output (${\dot{\text{V}}}$CO_2_), and respiratory rate (RR), or minute ventilation (${\dot{\text{V}}}$_E_) were measured. Regarding ${\dot{\text{V}}}$O_2_, ${\dot{\text{V}}}$CO_2_, and RR during the rest period and in each motion, ${\dot{\text{V}}}$O_2_ and ${\dot{\text{V}}}$CO_2_ in males were significantly greater than those in females. RR in the female participants had greater value than that in males. Among the maneuvers, ${\dot{\text{V}}}$O_2_, ${\dot{\text{V}}}$CO_2_ or ${\dot{\text{V}}}$_E_ during a hair-washing motion with both hands were greater than those during hair-washing motion with one hand. ${\dot{\text{V}}}$O_2_, ${\dot{\text{V}}}$CO_2_, RR, or ${\dot{\text{V}}}$_E_ during a hair-washing motion with both hands fast speed was greater than those during a hair-washing motion with slow speed. In conclusion, this study showed the effects owing to the differences in motion maneuvers and gender during UAE in healthy young people. These suggest a need to consider motion maneuver or gender when teaching motion methods of activities of daily living on the patients.

## Introduction

Chronic obstructive pulmonary disease (COPD) is characterized by airflow limitation. Patients with COPD frequently experience dyspnea during unsupported arm exercise (UAE). It is known that respiratory function is altered in the UAE during activities of daily living (ADL). Accordingly, patients with early-stage COPD already have significant ventilatory constraints and increased dyspnea during ADL^1,2^.

Patients with COPD regarded personal body care, including hair-washing, as a routine task with a relatively fixed form and a symbolic means of self-preservation^3^. Nevertheless, it is a motion that involves repetitive movements with UAE. Therefore, it is necessary to provide guidance on the motion that reduces dyspnea. Moreover, a few studies have examined the effects of differences in ventilatory parameters between one hand and both hands or fast and slow speed.

The previous studies have suggested differences in the respiratory function between males and females in maximal exercise^4–6^. However, these differences between males and females during low-intensity exercise with UAE are unclear.

In this study, we attempt to elucidate the effects of differences in the UAE and gender on respiratory variables in healthy people. A hair-washing motion was chosen for this study as ADL include UAE. The aim of this study was to investigate effects on respiratory variables during hair-washing motion across gender and motion type in healthy young people.

## Methods

### Subjects

A total of 33 healthy young people (21 ± 1 and 20 ± 1 year old for males and females, respectively) were enrolled in this study (characteristics are shown in Table 1). The inclusion criteria were nonsmoker and absence of cardiac and pulmonary diseases. All the participants gave written informed consent prior to the inclusion. Ethical approval for this study was granted by the Ethics Review Board of Yamagata Prefectural University of Health Sciences, Yamagata, Japan (#1801-23).

**Table 1:** Physiological characteristics of the participants (N = 33).

Characteristic	Males (N = 16)	Females (N = 17)
Age (years)	21 ± 1	20 ± 1
Height (cm)	170.2 ± 6.0	156.8 ± 5.0
Weight (kg)	63.5 ± 9.5	52.2 ± 7.3
BMI (kg/m^2^)	22.0 ± 2.8	21.2 ± 2.3

Data are presented as mean ± standard deviations.

*BMI* body mass index.

### Hair-washing motion measurements

**Figure 1:**
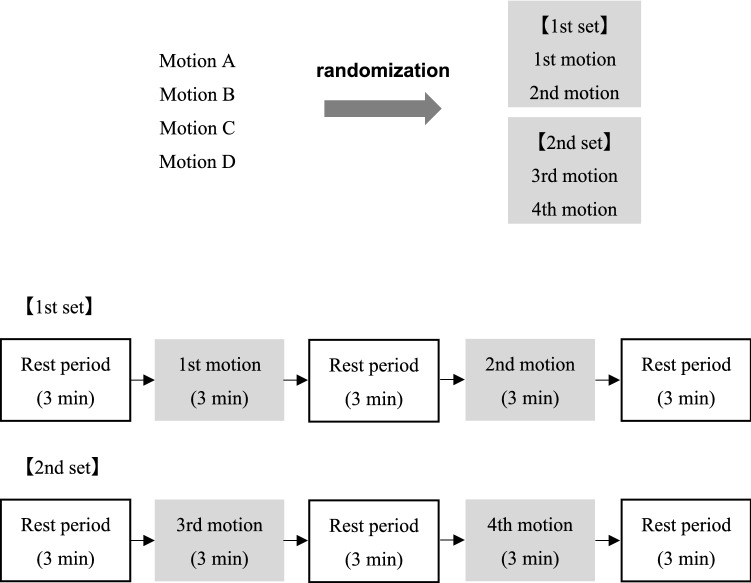
Measurement procedure of the hair-washing motion. Motion A, both hands with fast speed; motion B, both hands with slow speed; motion C, one hand with fast speed; motion D, one hand with slow speed.

In this study, the participants performed hair-washing motion to examine the influence in difference in maneuver or speed of motions. We defined a single trace of action from the top of the head to the back of the head. The participants performed the following 4 types of hair-washing motions, combining both hands and one hand at two speeds (60 times/min or 160 times/min): both hands with fast speed (motion A), both hands with slow speed (motion B), one hand with fast speed (motion C), and one hand with slow speed (motion D). These motions were divided into 2 sets and continued for 3 min with resting periods before and after. There was a break time between the two sets (Fig. 1). The order in which the motions were performed was randomly determined.

### The respiratory variable measurements

Respiratory variables such as oxygen uptake (${\dot{\text{V}}}$O_2_, ml / min, STPD), carbon dioxide output (${\dot{\text{V}}}$CO_2_, ml/min, STPD), minute ventilation (${\dot{\text{V}}}$_E_, l/min, BTPS), or respiratory rate (RR, bpm) were measured by breath-by-breath methods using a portable metabolic measurement system (AE-100i MINATO; Osaka, Japan), and heart rate (HR, bpm) was continuously measured using a heart rate monitor (Polar T31, Polar Electro, Finland). Oxygen (O_2_) and carbon dioxide (CO_2_) in the participant’s exhaled gas were detected by the analyzer’s O_2_ and CO_2_ meter. From these data, the device calculated ${\dot{\text{V}}}$O_2_, ${\dot{\text{V}}}$CO_2_, ${\dot{\text{V}}}$_E_, RR, etc. The response time for the O_2_ and CO_2_ analyzer was less than 250 ms (cited form instruction manual of MINATO). The measurement range of the flow sensor was 0–840 L/min, and its measurement accuracy was within 3% of the indicated value. The precision of this analyzer has been reported in a previous study^7^. A complete calibration routine was performed before each experiment. The respiratory variables were measured as standard temperature and pressure, dry. We used the mean value of the data for the last 1 min of each motion and each rest period. The mean value of the respiratory variables during the last 1 min of the first rest period was used as a baseline to compare the increases in the parameters for each motion.

### Statistical analysis

Means ± standard deviation was calculated for the female and male subgroups during the rest period and in each motion (motions A–D and baseline). The data were analyzed using IBM SPSS Statistics software, version 24.0 (IBM Corporation, Armonk, NY, USA). Student’s t test was used to analyze differences in respiratory variables between males and females. Three-way analysis of variance (ANOVA) with repeated measures was used to compare the effects across (1) gender and (2) motion (one hand or both hands), and speed (fast or slow) on respiratory variables. Bonferroni’s correction was used for the post-hoc analysis. The level of significance was set at p < 0.05 for all statistical comparisons.

### Ethical approval

This study was performed in line with the principles of the Declaration of Helsinki. Approval was granted by the Ethics Committee of Yamagata Prefectural University of Health Sciences, Yamagata, Japan (#1801-23).

## Results

Participants performed 4 type hair-washing motion (motion A: both hands and fast speed, motion B both hands and slow speed, motion C: one hand and fast speed, motion D: one hand and slow speed). Respiratory variables (${\dot{\text{V}}}$O_2_, ${\dot{\text{V}}}$CO_2_, HR, RR, and ${\dot{\text{V}}}$_E_) were measured in the rest period and each motion.

Mean values of each respiratory variables such as ${\dot{\text{V}}}$O_2_, ${\dot{\text{V}}}$CO_2_, HR, RR or ${\dot{\text{V}}}$_E_ during the rest period and each motion are shown in Table 2. Respiratory variables in each motion increased compared to those in rest period. In ${\dot{\text{V}}}$O_2_, ${\dot{\text{V}}}$CO_2_, HR, and ${\dot{\text{V}}}$_E,_ motion A showed the greatest value among 4 motions.

**Table 2:** Respiratory variables in the 4 different motions and rest period.

		Rest	Motion A	Motion B	Motion C	Motion D
${\dot{\text{V}}}$O_2_ (mL/m)	M	270.1 ± 74.2	351.3 ± 92.4***	306.6 ± 95.8**	294.2 ± 95.5	273.6 ± 96.2
	F	204.8 ± 45.5	258.1 ± 49.1***	231 ± 52.0**	228.5 ± 50.8	220.4 ± 48.0
${\dot{\text{V}}}$O_2_ (mL/kg/m)	M	4.2 ± 0.9	5.5 ± 0.9***	4.8 ± 1.1**	4.6 ± 0.9	4.2 ± 1.0
	F	3.9 ± 0.8	4.9 ± 0.7***	4.4 ± 0.9**	4.4 ± 1.0	4.2 ± 0.9
${\dot{\text{V}}}$CO_2_ (mL/m)	M	244.1 ± 75.8	352.0 ± 123.6***	297.3 ± 112.8**	305.7 ± 168.3	255.5 ± 102.6
	F	177.5 ± 37.9	249.2 ± 53.8***	216.9 ± 49.0***	210.5 ± 42.2**	203.7 ± 47.2**
HR (bpm)	M	71.5 ± 22.7	91.8 ± 10.7**	88.4 ± 10.9**	86.6 ± 11.2**	82.9 ± 10.6**
	F	77.8 ± 8.4	86.9 ± 24.7*	83.2 ± 26.2	80.5 ± 23.5	81.1 ± 23.5
RR (bpm)	M	15.6 ± 2.4	19.2 ± 4.6*	18.4 ± 4.4*	19.9 ± 4.3**	18.4 ± 3.6**
	F	17.7 ± 4.1	24.4 ± 6.7***	22.6 ± 6.3***	24.3 ± 6.0***	21.4 ± 5.4**
${\dot{\text{V}}}$_E_ (L/m)	M	9.2 ± 2.5	13.4 ± 3.5***	11.5 ± 3.2***	12.7 ± 6.5*	10.4 ± 3.0**
	F	7.5 ± 1.6	11.3 ± 2.3***	9.9 ± 2.2***	10.1 ± 2.1***	9.3 ± 2.1***

All data are expressed as mean ± SD.

Motion A, both hands with fast speed; motion B, both hands with slow speed; motion C, one hand with fast speed; motion D, one hand with slow speed.

*M* males, *F* females.

### Effects of gender, motion and speed on respiratory variables during a hair-washing motion

Effects of gender, motion and speed on respiratory variables (${\dot{\text{V}}}$O_2_, ${\dot{\text{V}}}$CO_2_, HR, RR and ${\dot{\text{V}}}$_E_) during a hair-washing motion were analyzed by three-way ANOVA and post-hoc analysis.

**Figure 2:**
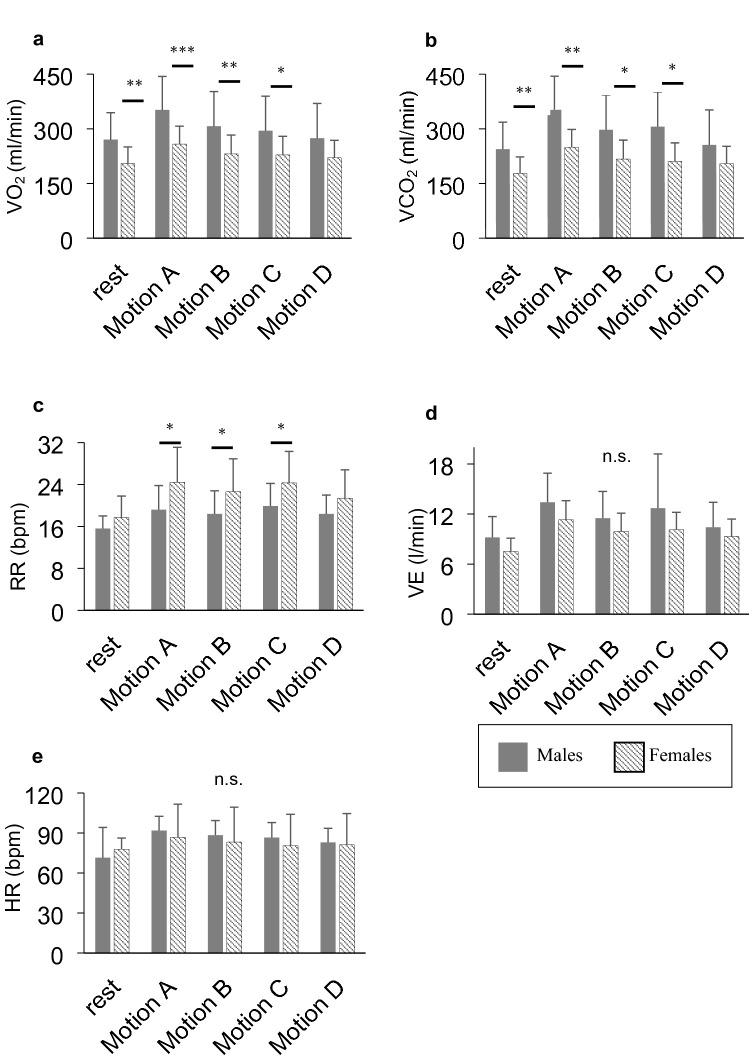
Difference between males and females. **(a)** ${\dot{\text{V}}}$O_2_; **(b)** ${\dot{\text{V}}}$CO_2_; **(c)** RR; **(d)** ${\dot{\text{V}}}$_E_; **(e)** HR. Motion A, both hands with fast speed; Motion B, both hands with slow speed; Motion C, one hand with fast speed; Motion D, one hand with slow speed. *p < 0.05, **p < 0.01, *** p < 0.001, n.s., not significant. ${\dot{\text{V}}}$O_2_, ${\dot{\text{V}}}$CO_2_, and RR showed differences between males and females. In contrast, ${\dot{\text{V}}}$_E_ and HR showed no significant differences by gender.

There was significant main effect of gender on ${\dot{\text{V}}}$O_2_, ${\dot{\text{V}}}$CO_2_, and RR (${\dot{\text{V}}}$O_2_: F_1,31_ = 8.83, p < 0.05; ${\dot{\text{V}}}$CO_2_: F_1,31_ = 6.93, p < 0.05; F_1,31_ = 6.03, p < 0.05). Post-hoc analysis showed ${\dot{\text{V}}}$O_2_ and ${\dot{\text{V}}}$CO_2_ in males was significantly greater than those in the female during rest period and motion A–C, RR in females was significantly greater than those in males during motion A–C (Fig. 2). ${\dot{\text{V}}}$O_2_ divided by body weight did not show a significant main effect of gender. These results indicated that each respiratory variable was affected differently owing to gender during hair-washing motion.

When considering the differences in the maneuver of motion, there was a significantly main effect of motion on ${\dot{\text{V}}}$O_2_, ${\dot{\text{V}}}$CO_2_, HR, ${\dot{\text{V}}}$_E_ (${\dot{\text{V}}}$O_2_: F_1,31_ = 53.00, p < 0.05; ${\dot{\text{V}}}$CO_2_: F_1,31_ = 56.36, p < 0.05, HR: F_1,31_ = 5.52, p < 0.05, ${\dot{\text{V}}}$_E_: F_1,31_ = 13.10, p < 0.05), and there was significantly main effect of speed on ${\dot{\text{V}}}$O_2_, ${\dot{\text{V}}}$CO_2_, RR, ${\dot{\text{V}}}$_E_ (${\dot{\text{V}}}$O_2_: F_1,31_ = 25.09, p < 0.05; ${\dot{\text{V}}}$CO_2_: F_1,31_ = 16.16, p < 0.05, RR: F_1,31_ = 11.01, p < 0.05, ${\dot{\text{V}}}$_E_: F_1,31_ = 15.91, p < 0.05). ${\dot{\text{V}}}$O_2_, ${\dot{\text{V}}}$CO_2_, HR, and ${\dot{\text{V}}}$_E_ during a hair-washing motion with both hands were greater than that during hair-washing motion with one hand. ${\dot{\text{V}}}$O_2_, ${\dot{\text{V}}}$CO_2_, RR, ${\dot{\text{V}}}$_E_ during hair-washing motion with both hands fast speed were greater than those during a hair-washing motion with slow speed.

### The relationship between gender, and motion or speed in ${\dot{\text{V}}}$O_2_

**Figure 3:**
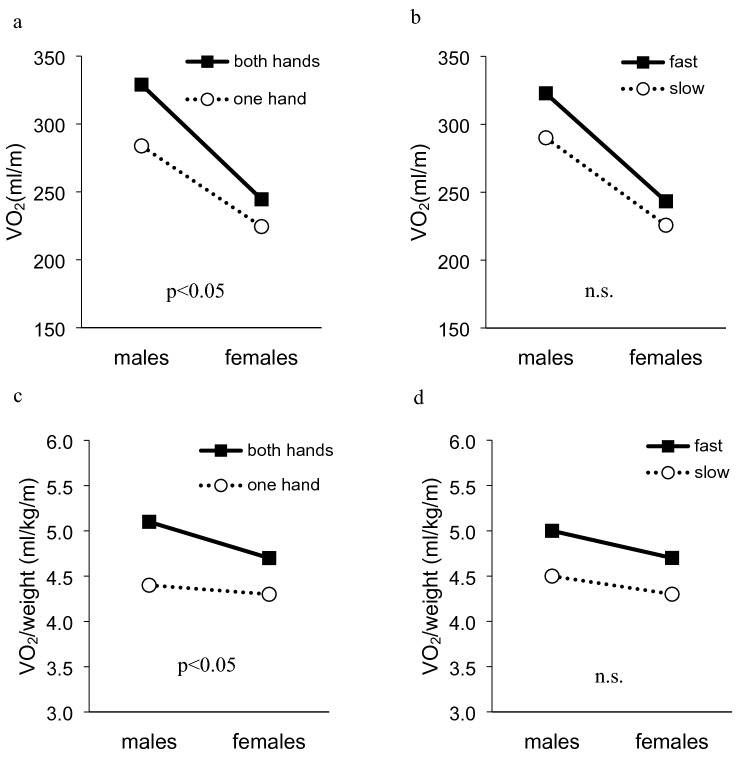
The relationship between gender and motion effects in VO_2._ There were significantly interactions between gender and motion. (**a, b**) Interaction in oxygen uptake, **(c, d)** interaction in oxygen uptake divided by body weight. n. s.: not significant.

A significant interaction between gender and motion was observed in ${\dot{\text{V}}}$O_2_ and ${\dot{\text{V}}}$O_2_/weight (F_1,31_ = 7.83, p < 0.05; F_1,31_ = 5.40, p < 0.05, respectively) (Fig. 3). On the other hand, there was no significant interaction between gender and speed in ${\dot{\text{V}}}$O_2_ and ${\dot{\text{V}}}$O_2_/weight. In ${\dot{\text{V}}}$CO_2_, RR, ${\dot{\text{V}}}$_E_, and HR, showed no significantly interactions among gender, motion and speed.

## Discussion

In this study, we attempt to elucidate the effects of the differences across gender and motion types for healthy young people by comparing ${\dot{\text{V}}}$O_2_, ${\dot{\text{V}}}$CO_2_, RR, HR, and ${\dot{\text{V}}}$_E_ during the performance of hair-washing motion. The main result of this study is that oxygen uptake and ventilation are significantly affected by the maneuvers of hair-washing motion and gender.

### Characteristics of hair-washing motion

Hair-washing motion requires UAE, that cause significant metabolic and ventilatory demands^8^. When an arm is elevated and unsupported, healthy people and patients with COPD have a significant increase in functional residual capacity^9^ and a reduction in inspiratory capacity^10^. Furthermore, the abdominal muscles stabilize the torso during arm exercise, and these activities decrease the end expiratory lung volume^8,11^. Therefore, tidal volume (${\dot{\text{V}}}$_T_) may be restricted and RR may increase to supplement oxygen demand during UAE. The mobilization of the sternocleidomastoid muscle during upper arm elevation suggests a decreased respiratory function^12^. These possibly cause dyspnea during upper arm elevation. The present study revealed that respiratory variables during each hair-washing motion was increased compared to the rest period. These finding indicated that UAE during ADL caused increasing ventilatory demands in healthy young people.

### Effects of gender

In the rest period, ${\dot{\text{V}}}$O_2_ and ${\dot{\text{V}}}$CO_2_ in males were significantly greater than those in females. It has been known that males have a greater oxygen uptake than females in the rest period, so observed differences in ${\dot{\text{V}}}$O_2_ between males and females might be affected by their muscle mass. In motion A–C, ${\dot{\text{V}}}$O_2_, and ${\dot{\text{V}}}$CO_2_ in the males were significantly greater than those in females. On the other hand, ${\dot{\text{V}}}$O_2_ divided by body weight showed no significant effect on gender. This result shows that differences in body weight between males and females affect the differences in ${\dot{\text{V}}}$O_2_. Previous studies suggested that differences between males and females for peak ${\dot{\text{V}}}$O_2_ depend on the size of the contracting muscle mass^13,14^. In this study, differences between the males and females in ${\dot{\text{V}}}$O_2_ and ${\dot{\text{V}}}$CO_2_ during each motion were observed. This result indicated that ${\dot{\text{V}}}$O_2_ during the hair-washing motion is affected by gender owing to the differences in contracting muscle mass, as well as during maximal exercise.

In the females, RR during the rest period and each motion was significantly greater than that in their males. On the other hand, ${\dot{\text{V}}}$_E_ during rest period and each motion showed no main effect on gender. This result suggests that chest kinematics may be significantly influenced by gender as revealed by the differences in the ventilatory values during the performance of hair-washing motion. ${\dot{\text{V}}}$_T_ and ${\dot{\text{V}}}$_E_ in females are mechanically constrained during exercise, since the lung volume of females is smaller than that of males^4,15^. Bellemare et al. suggested that the rib cage muscle contributes more to the inspiratory pressure swings in females than males. Altogether, females respond to increased oxygen demand by increasing their RR with their rib cage muscle efforts.

Previous study showed differences on respiratory variables between males and females during maximal exercise^16–18^. This study suggested that there are differences in respiratory function during UAE between males and females, even though motions did not require maximal effort for healthy young people. Further studies are need to reveal the effects of gender during ADL with UAE for elderly people and patients with respiratory diseases.

### Effects of maneuver of motion

The results of this study show significant increases in the ventilatory parameters, such as ${\dot{\text{V}}}$O_2_, ${\dot{\text{V}}}$CO_2_, RR, HR, or ${\dot{\text{V}}}$_E_, during the performance of motion with both upper arms. Also, the speed of motion may have influenced these ventilatory parameters.

Motion A and B required elevation of both upper limbs, motion C and D required elevation of one limb. Motion A and C were performed at fast speed compared with motion B and D. Therefore, the results showed the highest values in ${\dot{\text{V}}}$O_2_ and ${\dot{\text{V}}}$CO_2_ during motion A. Noteworthy, the increase in ${\dot{\text{V}}}$O_2_ in males was greater than that in females when comparing the motion with one hand and both hands. Motion with both hands increased oxygen uptake. Therefore, males might be effect on motion with one hand or both hands compared with females. This result extends our knowledge of effects of differences motion and gender during the UAE.

When the participants performed hair-washing motion with fast speed, the waveform amplitude of their inferior fibers of the trapezius increased^19^. This suggests that the hair-washing motion with fast speed increases oxygen demand compared with hair-washing motion with slow speed.

In the results of this study, RR increased on fast-speed motion compared with slow speed motion. We deduced that the increase in RR was caused by two factors. One of these is limited ventilation owing to upper arm elevation, and another is locomotor–respiratory coupling (LRC). It is known that LRC is initiated between exercise rhythm and RR during exercise^20,21^. LRC refers to the phase-locking of locomotor and respiratory frequencies. The mechanism underpinning LRC has not been elucidated yet, but LRC has been suggested to increase respiratory airflow^22^. In this study, we could not confirm that the hair-washing motion with fast speed caused LRC. Nevertheless, it is possible to suggest that LRC occurs with fast-speed motion owing to the action of relieving the respiratory load caused by the UAE.

Hair-washing motion with one hand and slow speed showed the lowest value in each motion for 3 min. This study suggests that we should choose motion maneuvers considering to factors such as gender and physical function.

## Conclusion

We showed the effects owing to the differences in motion maneuvers and gender during the UAE in healthy young people. The findings from this study made several contributions to reveal changes of respiratory demands during ADL. These suggest a need to consider motion maneuver or gender when teaching motion methods of ADL on the patients.
